# The Magic Staff: A Comprehensive Overview of Baculovirus-Based Technologies Applied to Human and Animal Health

**DOI:** 10.3390/v15010080

**Published:** 2022-12-28

**Authors:** Matías L. Pidre, Paula N. Arrías, Leslie C. Amorós Morales, Víctor Romanowski

**Affiliations:** Instituto de Biotecnología y Biología Molecular (IBBM), Universidad Nacional de La Plata (UNLP) and Consejo Nacional de Investigaciones Científicas y Técnicas (CONICET), La Plata 1900, Argentina

**Keywords:** baculovirus, BEVS, BacMam, viral vectors, vaccines, gene therapy

## Abstract

Baculoviruses are enveloped, insect-specific viruses with large double-stranded DNA genomes. Among all the baculovirus species, *Autographa californica multiple nucleopolyhedrovirus* (AcMNPV) is the most studied. Due to its characteristics regarding biosafety, narrow host range and the availability of different platforms for modifying its genome, AcMNPV has become a powerful biotechnological tool. In this review, we will address the most widespread technological applications of baculoviruses. We will begin by summarizing their natural cycle both in larvae and in cell culture and how it can be exploited. Secondly, we will explore the different baculovirus-based protein expression systems (BEVS) and their multiple applications in the pharmaceutical and biotechnological industry. We will focus particularly on the production of vaccines, many of which are either currently commercialized or in advanced stages of development (e.g., Novavax, COVID-19 vaccine). In addition, recombinant baculoviruses can be used as efficient gene transduction and protein expression vectors in vertebrate cells (e.g., BacMam). Finally, we will extensively describe various gene therapy strategies based on baculoviruses applied to the treatment of different diseases. The main objective of this work is to provide an extensive up-to-date summary of the different biotechnological applications of baculoviruses, emphasizing the genetic modification strategies used in each field.

## 1. Introduction

The baculovirus expression vector system (BEVS) has been around since the early 1980s. Originally, these insect-specific pathogens were attractive because of their usability as biological pest control agents, posing as an alternative to chemical insecticides in the agroindustry. However, with the successful production of recombinant human IFN-β [1] by employing genetically-modified *Autographa californica* multiple nucleopolyhedrovirus (AcMNPV) and lepidopteran cells, BEVS positioned itself as a highly versatile and powerful tool in biotechnology, and to this day thousands of proteins have been expressed in this system, some of them even becoming commercially available.

**Figure 1:**
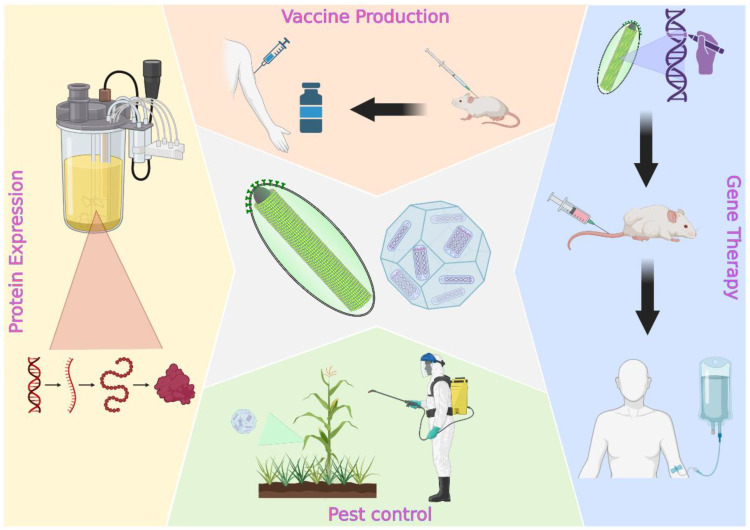
Schematic representation of main applications for baculovirus-based technologies. Created with BioRender.com, (accessed on 29 November 2022).

BEVS comprises an attractive platform because it is significantly safer than other viral-based technologies that employ mammalian cell culture, and also because of the lower production costs and, most importantly, its versatility. It is this last characteristic that has made BEVS grow exponentially, and it is the reason why biotechnological companies and research labs are choosing it over more traditional alternatives. Here, we will provide an overview of how BEVS can be applied to human and veterinary health, focusing on the different designs that can be used to obtain immunogens and gene therapy vectors (Figure 1). An extensive literature search was carried out using electronic databases: PubMed, Scopus, Science Direct and Google Scholar. Some combinations of keywords used to collect the information used for writing the review were: baculovirus, baculovirus molecular biology, baculovirus expression vector, baculovirus vaccines, baculovirus and virus-like particles, baculovirus display, baculovirus and gene therapy, baculovirus and adeno-associated viruses, BacMam, among others. All the articles were selected based on their scientific relevance and more recent publications were privileged.

## 2. Baculovirus in Nature

The Baculoviridae family is composed of viruses that infect arthropods belonging to the orders Lepidoptera, Hymenoptera, Diptera, Orthoptera, Coleoptera, Neuroptera, Thysaneura and Trichoptera [2] in their larval stages. Baculoviruses have circular, double-stranded DNA genomes of around 80 to 180 kbp, which are packed inside a rod-shaped nucleocapsid which resembles a staff. The size of this nucleocapsid is roughly 40–50 nm in diameter and 200–400 nm in length and it is enveloped in a membrane derived from the host cell. 

Currently, the taxonomic classification of Baculoviridae includes four genera; *Alphabaculovirus*, *Betabaculovirus*, *Gammabaculovirus* and *Deltabaculovirus.* The model baculovirus; *Autographa californica* nucleopolyhedrovirus (AcMNPV) belongs to the *Alphabaculovirus* genus and has been widely studied since its discovery. Even though biotechnological applications have been described based on other baculoviruses, such as Bombyx mori nucleopolyhedrovirus (BmNPV) and its famous host, the silkworm [3,4], this review focuses mainly on AcMNPV and its applications.

The nucleocapsid is incorporated into two different types of virions, depending on the stage of the life cycle. Outside the host, the virions are found embedded in a protein matrix forming the characteristic occlusion bodies (OBs) that protect the viruses from rough environmental conditions such as UV radiation, and desiccation, among other factors. The viral cycle of baculoviruses starts when a susceptible insect feeds on foliar material containing OBs, and the protein matrix disintegrates in the midgut of the insect due to the change in pH, releasing the so-called occlusion-derived viruses (ODVs) that infect the epithelial cells of the larval intestine via a membrane fusion mechanism. 

The infected cells produce a second type of virion, designated budded-virus (BV), which spreads infection within the body of the insect larva [2,5].

Gene expression is temporally regulated in the form of a transcriptional cascade, where gene products from early stages regulate the production of the later ones. Early genes (immediate early class) are transcribed by the action of a host RNA polymerase, while the late and very late genes require a viral-encoded polymerase.

During late stages of infection, the virus progeny switches from producing BVs to retaining nucleocapsids within the nucleus, envelopment and inclusion into a virally encoded protein (polyhedrin) matrix to build multiple OBs (polyhedra), which are later released into the environment after cell lysis and liquefaction of the insect larva.

These characteristics inherent to the natural infection cycle of baculoviruses, in addition to their narrow host range, have made them an important bio-input tool for integrated pest management [6].

## 3. Budded Viruses and Cell Lines

Although the use of baculoviruses as biological pest control agents takes advantage of the OBs, most of the biotechnological applications of baculoviruses utilize the BVs. 

BVs are the morphotype that can be propagated and employed in cell culture. The virions are enveloped and present a class III glycoprotein in their coats, called GP64. GP64 is responsible for the attachment and entry of the virus inside the host cell via interaction with a not-very-well-characterized cellular receptor. 

Even though baculoviruses have a very limited host range in terms of their pathogenicity and replication, budded viruses can successfully enter multiple types of cells. In terms of insect cell lines, those derived from *Trichoplusia ni* and *Spodoptera frugiperda* ovaries are the most widely employed. Commercially available cell lines include Sf9, Sf21, and High Five (BTI-Tn-5B1-4), among others. Even though insects are eukaryotes, the N-glycosylation patterns of these animals differ from those of mammals; while insects produce relatively simple N-glycans with terminal mannose residues, mammals have a wide variety of enzymes that result in more complex N-glycans with terminal sialic acid residues. Depending on the protein, N-glycosylation can be of extreme importance for its function. For this reason, genetically engineered cell lines have been developed which include, but are not limited to, cells that can add terminal N-acetylglucosamine, galactose, and sialic acid residues, but also cells that can prevent the addition of fucose residues to N-glycans [7]. On the same line, SweetBac [8] consists of a baculoviral genome that provides the enzymes required for the production of mammalian-like N-glycans and can be employed for the production of recombinant baculoviruses that express mammalian glycoproteins. PolyBac [9] is another engineered bacmid that allows multiple transgene insertions, and has been exploited for the customized modification of the glycosylation pathway in insect cells. A set of PolyBac-derived bacmids with different N-glycosylation enzymes has been produced, and these bacmids can be used to express a protein of interest together with the glycosylation enzymes that will provide the desired glycosylation pattern.

## 4. Recombinant Baculovirus Production

BEVS usually requires the generation of at least one recombinant baculovirus that carries the gene of interest (GOI). Although there are multiple commercially available systems for the production of recombinant baculoviruses, most of them are based on one of two strategies. The first one involves homologous recombination inside insect cells between a baculovirus genome engineered to replicate in *E. coli* (a bacmid), which carries all the genetic information for the generation of the virus but might be truncated on an essential gene so it cannot produce wild-type virus, and a transfer plasmid, which carries the GOI sequence downstream of an insect or baculovirus promoter. This expression cassette (promoter, GOI, and transcription termination signal) is flanked by two regions that will allow the recombination with the bacmid. When recombination occurs, the essential gene is reconstituted, restoring the ability of the baculovirus carrying the GOI to replicate in insect cells. Oxford Expression Technologies’ *flash*BAC™ platform employs this procedure. 

The other strategy involves the site-specific transposition of Tn7 transposon into a bacmid. The commercially available Bac-to-Bac Expression System (Invitrogen Inc.) is based on this approach. Briefly, it consists of the following: a transfer plasmid vector carrying the GOI under the control of a specific promoter flanked by Tn7 transposition sites that allow transposition inside *Escherichia coli* DH10Bac cells, which contain both the bacmid and a helper plasmid that codes for the Tn7 transposase. After the transposition in bacterial cells, the bacmid is purified and transfected into insect cells to generate the recombinant baculovirus.

For the generation of baculoviruses that carry more than one or two genes of interest, there are also systems available. MultiBac [10] is a modified baculoviral genome that allows the insertion of multiple GOI via iterative cycles of sequence and ligation-independent cloning coupled to Cre-mediated fusion of plasmids. More recently, Neuhold and colleagues [11] presented a golden-gate-based single-step cloning system called GoldenBac that is compatible with both Tn7 transposition and homologous recombination (HR) bacmids. Along the same lines, SmartBac [12], employs a polyprotein approach that is constructed by overlapping PCR and Gibson assembly coupled with Cre-LoxP recombination for the generation of large transfer plasmids that carry more than one GOI. 

It is important to note that a recent report by Jacob et al. [13] shows that HR systems for the generation of recombinant baculoviruses have increased stability when compared to the Tn7-based, which suggests that HR systems might be preferable for the production of safe biologicals. In addition, experiments have been also used to identify the minimal baculovirus genome and improve the construction of better expression vectors [14,15].

## 5. Baculovirus-Derived Immunogens for Vaccine Development

Generally speaking, a good immunogen in vaccinology should induce a response that will totally prevent or reduce pathogen infection.

The ability of an immunogen to produce a good correlate of protection will depend a lot on its properties; while those that imitate natural infection such as live attenuated strains of viruses and bacteria usually generate efficient vaccines, others such as subunit vaccines made out of polysaccharides are not as effective and might require more complex formulations that incorporate adjuvants and the vaccination schemes might be composed of multiple doses and boosters. For this reason, rational immunogen design is crucial, which includes studying functional epitopes, and key proteins, among others. 

The high versatility of the BEVS system has been broadly exploited for the production of a vast and diverse amount of immunogens both for human and veterinary uses.

### 5.1. Recombinant Proteins

When developing subunit vaccines, i.e., vaccines that do not carry full pathogens but only parts of them, the production of recombinant proteins is, more often than not, the chosen strategy. 

BEVS provides a fast, adaptable, and low-cost platform for the production of heterologous proteins, which is of utmost importance when dealing with global sanitary emergencies such as the COVID-19 pandemic. Some authors have reported [16] that in the case of influenza vaccines, employing BEVS instead of the classic embryonated egg platform, the time needed to produce 425 million vials of the vaccine would be 45 days instead of the average six months. FluBlok® [17] (Protein Sciences Corporation) is a quadrivalent influenza vaccine produced in BEVS by the expression of the hemagglutinin (HA) proteins of four different strains of influenza A and B viruses and is currently FDA-licensed for individuals 18 years old and older. 

In pre-clinical stages, Shin et al. successfully produced a recombinant vaccine candidate against Zika virus (ZIKV) [18]. This potential vaccine is based on the baculovirus-expressed envelope protein of two strains of ZIKV, and its protective ability was shown in pregnant mice and their offspring, indicating maternal antibody transmission to the fetuses. Moreover, they also observed cross-antibody-mediated neutralization of the dengue virus (DENV). Other viral antigens produced in the system have also shown good immunogenicity in pre-clinical stages, such as the mink circovirus (causative agent of mink refractory diarrhea) capsid protein [19], parvovirus VP2 protein [20], and VP4 and VP35 from grass carp reovirus (GCRV) [21], among others. It is important to note that the aforementioned proteins have been expressed in their wild-type form with no artificially built modifications. 

Furthermore, chimeras carrying the antigen of interest and another protein are also used. For example, Lee and collaborators [22] showed for the first time that a chimera composed of the vesicular stomatitis virus glycoprotein (VSV-G) and the VP1 GH loop epitope of foot-and-mouth disease virus produced in BEVS surpassed the commercially available vaccine in terms of immunogenicity in field studies. 

In addition to traditional vaccines that aim at preventing disease in the vaccinated individual, other types of vaccines can also be produced. A specific type of vaccine, called malaria transmission-blocking vaccines (TBVs) serves as an important accessory tool to accelerate the elimination of malaria. TBVs induce the production of antibodies that prevent the sexual stage of plasmodium from entering the mosquito midgut, and, in consequence, impair the transmission between humans and the insect. Recently, two reports of recombinant TBV candidates produced in BEVS were published. Lee et al. [23] showed that Pfs48/45 6C protein fragment expressed in BEVS does not require a fusion partner and that by preventing N-glycosylation, 6C protein is biologically active and exhibits significant transmission-reducing activity. The other report, by Qui and collaborators, showed that HAP2 from *Plasmodium vivax* expressed in BEVS displays transmission-blocking activity and they propose it for the first time as a novel TBV candidate [24].

Regarding bacterial diseases, such as diphtheria and tetanus, vaccines seek to target the toxins that are responsible for the disease. Toxoid vaccines are strictly subunit vaccines which are usually composed of a chemically inactivated form of the toxin or a recombinant form of it that has been rendered inactive. An effective example of a baculovirus-expressed toxoid is the production of a nontoxic form of the C-terminal CPA toxin of *Clostridium perfringens* [25].

Since the beginning of the COVID-19 pandemic, the development of immunogens for severe acute respiratory syndrome coronavirus 2 (SARS-CoV-2) has been the focus of many pharmaceutical companies, governmental institutions, and research laboratories alike. Despite the existence of insect-cell-produced immunogens in advanced stages of development, the first vaccines to be approved in the context of the emergency were produced in other systems. However, Novavax Inc. COVID-19 vaccine, sold today under the names Nuvaxovid and Covovax was the first BEVS-produced COVID-19 vaccine to gain FDA approval, and the fifth overall. Nuvaxovid is described by its manufacturers as a nanoparticle vaccine but can be classified as a subunit vaccine. It is composed of a modified version of the spike protein, with prolines substituting the original residues (K986P and V987P) to maintain the pre-fusion conformation of the glycoprotein, and make it resistant to proteolytic cleavage [26]. The protein produced in Sf9 cells is incorporated in the cell membranes, and then harvested and assembled into lipid nanoparticles, along with M-Matrix as an adjuvant. It has a reported overall efficacy of 90.4% in a two-dose scheme and a 100% efficacy for the prevention of moderate to severe disease [26]. 

### 5.2. Virus-like Particles 

In the previous section we described subunit immunogens, that even though they comprise a safer alternative to attenuated live vaccines, they tend to require more doses or boosters to elicit an effective immune response. A very attractive alternative for viral immunogens is virus-like particles (VLPs). VLPs are self-assembling nanostructures that can resemble the enveloped virions or the capsids of non-enveloped viruses. They can be composed of just one or multiple viral proteins, in their full length or truncated, wild-type or genetically engineered. In contrast to virions, VLPs are not replication-competent entities; although some genetic material might be present, they are not able to replicate, nor revert to pathogenic genotypes. Vaccines that are based on these structures combine the advantages of attenuated virions and subunit vaccines; their nanometric sizes, their self-adjuvating properties and the distribution of multiple copies of the epitopes can be effectively recognized by the immune system and stimulate it. 

Baculoviruses and insect-cells have been widely employed for the production of VLPs, using diverse approaches. The simplest one consists of the infection of susceptible insect cells with a recombinant baculovirus that carries the wild-type version of the immunogen that can self-assemble into VLPs. Depending on the nature of the protein, the VLPs can be harvested from the supernatant (enveloped VLPs, that bud directly from the cell membrane) or from a cell-lysate (capsid VLPs). Examples of this strategy include VLPs derived from HIV gp140 (gp120 and the ectodomain of gp41) [27], chimeric norovirus VP1 [28] and wild-type norovirus VP1 [29], bovine papillomavirus type 9 (BPV9) L1 [30], coxsackievirus B4 VP1 [31], among others. All of these VLPs were found to be immunogenic in pre-clinical stages. 

More complex VLPs, i.e., those carrying multiple proteins, can also be produced by recombinant baculovirus infection of susceptible cells or larvae. For this design, we can find two alternatives; co-infection with multiple baculoviruses, each carrying only one foreign protein gene or infection with a single baculovirus that carries the genes of interest under the control of different promoters. Using the latter approach, Bernardino and collaborators [32] successfully generated rabies VLPs that carry the envelope protein (RVGP) and the matrix protein (RVM). On the same line, Zhang et al. [33] generated coxsackievirus B5 (CVB5) VLPs that contain VP1 and VP3 capsid proteins. These VLPs mimicked the shape and size of CVB5 virions and were found to induce a more robust immune response, measured as the total IgG levels and the neutralizing antibodies, compared to the one induced by the inactivated vaccine that conferred protection in mice challenged with a pathogenic strain of CVB5.

The co-infection strategies, although able to produce complex VLPs, require optimization of multiplicities of infection (MOI) to determine the best combination in order to produce proper stoichiometric incorporation of the antigens of interest into the VLPs, which is not always straightforward. However, some multi-protein VLPs are easier to produce, when only one recombinant baculovirus is required. This is the case for VLPs derived from viruses that encode polyproteins that are self-cleavable. Multiprotein VLPs of this type have been produced in insect cells for dengue virus [34] and enterovirus 71 [35], among others.

A downside to these two strategies is the fact that VLPs need to be purified and separated from budded baculoviruses (BVs). Although BVs themselves can act as adjuvants and promote a T CD8+ immune response, maturation of dendritic cells and subsequent production of pro-inflammatory cytokines [36,37], some formulations may require to be free of them, and this involves an extra purification step.

An alternative to infection-mediated strategies is the development of stably transformed recombinant insect cell lines. There are commercially available vectors for this, such as pIB/V5-His and its variations (ThermoFisher Scientific), but the vectors can also be constructed *in-house*. This alternative is BV-free and facilitates purification, which is especially important in the case of enveloped VLPs. For example, Khanefard and colleagues [38] successfully generated a transgenic Sf9 cell line that produces H5N1 influenza neuraminidase VLPs in a constitutive manner and are immunogenic. 

Commercially available VLP vaccines have been around for some time now (the first recombinant protein vaccine for HBV was licensed in 1986) [39], but Cervarix® (GlaxoSmithKline), which shows protection against different types of human papillomaviruses (HPV16 and HPV18), remains the only one produced in insect cells and approved for human use. Cervarix® is composed of L1 proteins from HPV types 16 and 18 [40,41], which are responsible for 70% of cervical cancers worldwide. New generation VLP-based HPV vaccines exhibiting broader protection spectra have been developed expressing both L1 and L2 proteins or L1-L2 chimeras [42] and multiple VLP-based vaccines for other pathogens are currently in advanced clinical trial stages. Currently, anti-SARS-CoV-2 vaccines are in different stages of development based on VLPs [43].

Although VLPs comprise a very attractive platform for the production of new-generation immunogens because they combine the advantages of inactivated and subunit vaccines, their purification can be tedious since the co-purification of impurities with the VLPs is common, which increases downstream processing costs. 

### 5.3. Baculovirus Surface and Capsid Display

The display of proteins of interest in the surfaces of viruses has multiple applications that range from the determination of protein–protein interactions to diagnostic tools as well as immunogens.

Baculoviruses as display vectors are interesting because in contrast to other viral vectors, there is no pre-existent immunity in humans and other vertebrate animals.

The display of a protein of interest on the surface of the budded virus, a process that is called surface display, can be achieved using different strategies which involve the production of chimeric fusions of the protein of interest or a specific peptide to GP64 or parts of it. Fusion of proteins to full GP64 results in chimeric proteins that possess all the signals required for transport to the membrane, maturation, and incorporation into the budded virus. Using this strategy, it has been possible to expose large proteins such as GP120 of HIV [44] and ROP4 of *Toxoplasma gondii* [45].

Another possible combination involves the fusion of the protein to the signal peptide (SP) of GP64, the transmembrane region (TM), and the cytoplasmic domain (CTD), in which the ectodomain of GP64 is replaced by the protein of interest. For example, Xue and collaborators successfully displayed varicella-zoster virus (VZV) glycoprotein E [46], which resulted in a strong immune response in mice. Moreover, the display of porcine circovirus 2 (PCV2) Cap(d41) protein was able to induce an immune response in both mice and pigs [47]. Using the same strategy, Luo and collaborators [48] generated a chimera of Zika virus (ZIKV) E protein that is displayed on the surface of BVs and provided protection against ZIKV in a mouse model. Although it is more common to display the ectodomain of membrane proteins fused to the TM of GP64, it is also possible to keep the wild-type TM of the protein of interest but fuse it to both the SP and the CTD of GP64.

In some cases, however, the protein can be incorporated into the envelope without fusion to GP64, as it happens for hemagglutinin (HA) [49] of influenza viruses or the protein G of vesicular stomatitis virus (VSV) [50,51]. 

It is important to mention that the recombinant baculoviruses still retain a wild-type copy of GP64, which will be incorporated into the envelope alongside the chimeric one.

Although baculovirus surface display can induce humoral responses, it is not very effective in inducing T CD8+ cellular responses [52,53], which are important for intracellular pathogen clearance. However, budded viruses can be useful tools for another type of antigen display, termed capsid display, that is more appropriate for the generation of this type of response since the translated protein is processed and can be presented via the MHC-I pathway.

Capsid display takes advantage of the ability of the baculovirus to enter a vast number of different cells, and it involves the incorporation of the protein of interest into the capsid of the BV. The principle is similar to surface display, and it involves the fusion of the protein or peptide of interest to the major capsid protein, VP39 [54]. 

It was demonstrated that fusion of OVA (ovalbumin) to VP39 allows MHC-I presentation of the antigen by professional presenting cells and the induction of cytotoxic immune responses [52,55] and that this strategy significantly reduces the amount of antigen required to elicit a strong immune response. 

## 6. Baculovirus Entry into Mammalian Cells: Transduction 

As mentioned before, although AcMNPV does not replicate in vertebrates, it can enter mammalian cells and express heterologous genes with mammalian promoters, a process known as gene transduction. The ability of baculoviruses to transduce mammalian cells has been used to induce specific immune responses [56] and, more recently, for the development of different gene therapy strategies. However, the entry mechanism of AcMNPV into mammalian cells is still unclear. Initially, it was suggested that electrostatic interactions between both viral and cell membranes played a crucial role in viral entry [57]. It was demonstrated that baculovirus interacts with heparan sulfate proteoglycans, more specifically syndecans, present on cell membranes, to bind and transduce mammalian cells [58]. Based on the mechanism of membrane fusion mediated by GP64 glycoprotein and the route of entry of baculovirus into insect cells, it was proposed that endocytosis mediated by clathrin, in a low pH-dependent manner, is the primary mechanism of entry in mammalian cells. Moreover, it was observed that virus fusion in early endosomes seems to be the major obstacle for efficient transduction [59]. Interestingly, it has been reported that caveolae may be involved in baculovirus entry into mammalian cells, as transduction efficiency was increased in the BHK21 cell line in the presence of genistein, an inhibitor that interferes with the formation of caveosomes [60]. On the other hand, some results indicate that baculoviruses can enter certain mammalian cell lines, such as hepatocytes, through a mechanism different from clathrin-mediated endocytosis or macropinocytosis, suggesting that phagocytosis might also be involved [61].

Although the entry mechanism of AcMNPV seems to vary in a cell-type-dependent fashion and requires further investigation, the relevance of the GP64 glycoprotein in mediating viral entry is very clear [62]. GP64 is crucial for viral attachment, internalization, and endosomal membrane fusion during baculovirus entry into both mammalian and insect cells [63]. The attachment of AcMNPV begins when GP64 interacts with molecules present on the cell surface and a change of pH inside the endosome triggers a conformational change of the glycoprotein. Once inside the cell, viruses are transported to the endosome, the nucleocapsids are released into the cytoplasm and they are immediately driven to the nucleus by actin-based motility enabling translocation through nuclear pore complexes [64]. In the nuclei, viral DNA is recognized and transcribed by host machinery; alteration of the cellular chromatin distribution was observed during this stage [65].

### Strategies to Improve Baculovirus Transduction Efficiency

**Figure 2:**
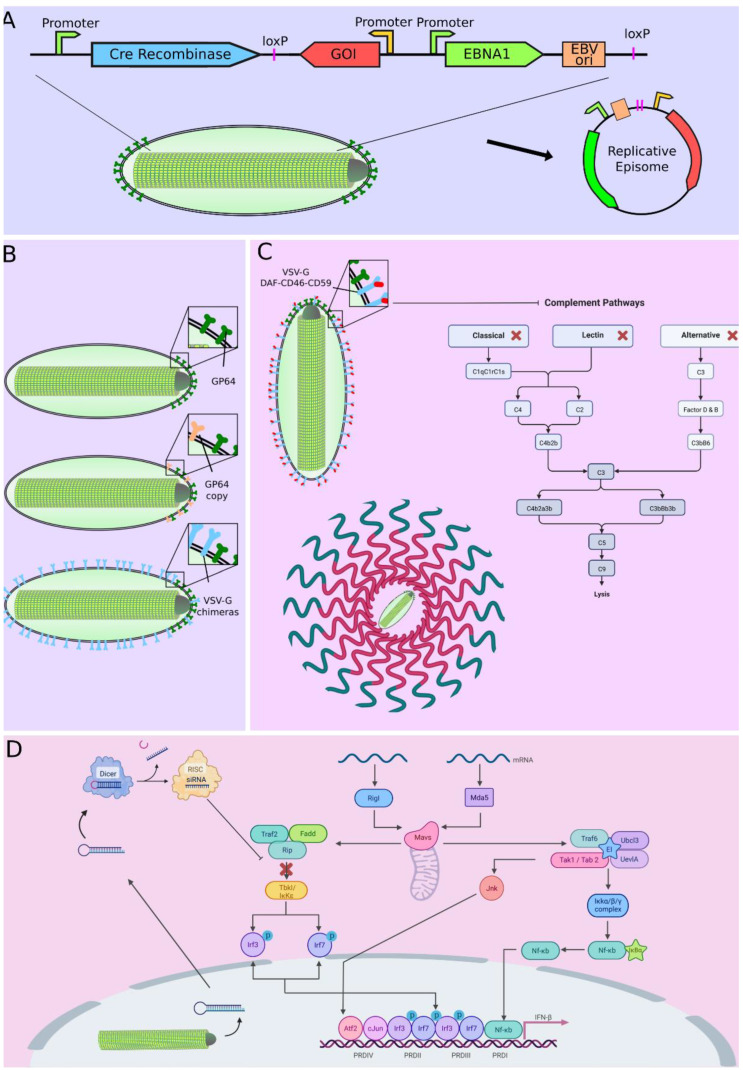
Improving transduction efficiency using vectors that produce replicative episomes (**A**), multiple copies of GP64 or VSV-G chimeras surface display (**B**), complement inhibitors surface display (**C**) and shRNA expression targeting antiviral response pathways (**D**). Adapted from “Detecting Viral Invaders” and “Complement Pathways”, by BioRender.com (accessed on 29 November 2022). Retrieved from https://app.biorender.com/biorender-templates.

Although baculoviruses can enter multiple types of cells, this process is not always efficient. In recent years, multiple strategies have been developed to improve baculovirus transduction efficiency. Some of them involve the modification of electrostatic interactions between virus and cellular membrane using chemical agents such as polyethyleneimine (PEI) or polyethylene glycol (PEG) [66,67]. Another method involves the pseudotyping of BV with fusion proteins of other viruses. In addition, it was reported that pseudotyped viruses with the G protein of vesicular stomatitis virus (VSV-G) were more efficient to transduce mammalian cells than those without pseudotyping (Figure 2B). Interestingly, no improvements in transduction efficiency were observed when a truncated version, instead of full-length VSV-G, was used at low multiplicity of transduction [68]. Furthermore, other proteins have been used to pseudotype baculoviruses, including influenza A virus neuraminidase [69] or the glycoprotein of Thogotovirus [70].

VSV-G has also been fused to tumor ligands (LyP-1, F3, and CGKRK) on the baculovirus surface, improving tumor binding and specific transgene expression two- to five-fold [71]. Another strategy to increase transduction efficiency involves the generation of avidin-displaying baculoviruses. This surface modification results in a significant increase in viral entry and, consequently, gene delivery [72]. However, not only viral proteins can increase transduction efficiency. Three malaria circumsporozoite protein variants have also been added to the baculovirus envelope to enhance transduction efficiency in hepatocytes [73]. The efficiency of transduction, however, does not only depend on cell entry, but also on the overall ability to express the transgenes. One strategy to increase baculoviral transduction efficiency, by improving transgene expression, consists of the suppression of genes involved in the antiviral response. Wang et al. reported that knockdown of the receptor-interacting protein kinase 1 (RIPK1), which plays an important role in immune response and regulates cell death, produced an increase in baculovirus-mediated transgene expression in different human cell lines (Figure 2D), without affecting cell viability or viral entry [74]. 

## 7. Gene Therapy

Gene therapy comprises a set of therapeutic strategies with great versatility since it can be adapted to each person to treat a variety of diseases, including cancer. There are two general categories into which gene therapy vectors can be classified: non-viral and viral vectors. Non-viral vectors consist mostly of conjugated polycationic polymers that allow the delivery of DNA. Positively charged liposomes are an example of this type of vector. Although these vectors are advantageous in biosafety, their application is restricted by low efficiency in transgene delivery and expression [75]. On the other hand, viral vectors, such as baculoviral, retroviral, lentiviral, adenoviral, and adeno-associated viral (AAV) vectors, have higher efficiency in cell entry and transduction by expressing different transgenes. The advantages and disadvantages depend on each particular viral vector [76]. Currently, AAV vectors, lentiviruses, and retroviruses have been successfully implemented and represent 19 FDA-approved gene therapy products [77].

However, lentiviral and retroviral vectors have low cloning capacity and there is a possibility of mutagenesis due to integration into the host genome. In addition, there are potential safety concerns for the development of replication-competent retroviruses [78]. The high cost of production, low scalability, and potential biosafety issues associated with current viral vectors highlight the great potential of using baculoviruses in gene therapy [79,80].

Due to their versatility and low toxicity, baculovirus gene delivery systems allow administration at specific sites, as well as the design and cloning of large complex therapeutic genes, mitigating adverse effects and improving therapeutics [81]. This gene therapy system is easily modifiable and could become a valuable tool for targeted gene therapy. Baculoviruses (BV phenotype) have already been used in several successful studies, including cancer treatment, vaccines, and regenerative medicine, showing their versatility [81,82,83,84].

In comparison with other common viral vectors, baculoviruses possess several advantages. In the first place, baculovirus-mediated transduction does not present any toxic effect against mammalian cells and does not disturb cell growth even at high MOI [85,86]. Furthermore, baculoviruses do not replicate in transduced mammalian cells [79]. These features of baculoviruses are particularly important because other viral vectors are human pathogens or integrate in the host genome and consequently represent a biological risk.

Another advantage of baculoviruses as gene therapy vectors consists of their large cloning capacity. The baculovirus (AcMNPV) genome is a 130 kb circular dsDNA molecule and has a maximum cloning capacity of at least 38 kb. This flexibility is particularly advantageous when compared to the limited cloning capacities of retroviral and AAV vectors [87].

Compared to other viral vectors, baculoviruses are easy to produce. Retroviral, lentiviral, and AAV vectors require the transfection of plasmids encoding essential genes into packaging cells for their production. In contrast, baculoviruses can be propagated by infecting insect cells in suspension or monolayer cultures and collecting the supernatant 3 to 4 days after infection. In addition, the whole process of construction, propagation, and handling of baculoviruses can be carried out in Biosafety Level (BSL) 1 laboratories without the need for specialized equipment. However, depending on the nature of the transgene included in its genome, biosafety levels higher than BSL-2 may be required.

Finally, one of the most important advantages is that mammals do not have pre-existing immunity against baculoviruses. In contrast, one of the most common problems associated with other viral vectors is that most people are exposed to these viruses during their lifetime and develop specific humoral responses. Then, circulating antibodies can significantly reduce the efficiency of viral vector transduction [87].

However, baculoviruses also have disadvantages as gene therapy vectors. One of these is that they induce transient transgene expression in mammalian cells. In vivo, transgene expression typically declines by day 7 and disappears by day 14 to 21 [81,88]. Moreover, the duration of in vitro transgene expression using baculoviruses is significantly shorter than expression mediated by retroviral, lentiviral, and AAV vectors.

Baculoviral vectors differ from other viral vectors in the time that the carried genes can persist in the host nucleus. DNA from retroviral, lentiviral, and adenoviral vectors can remain in the nucleus either in an integrated or episomal form, for long periods. In contrast, Tjia et al. demonstrated that baculoviral DNA persists in the nuclei of transduced mammalian cells for only 24–48 h [76].

Another disadvantage of using baculoviruses as gene therapy vectors is the inactivation by complement. The contact between baculoviruses and serum complement results in rapid inactivation of BVs. Several modifications are needed to reduce the negative effect of complement on baculovirus-mediated transduction. However, the complement system is not a problem only for baculovirus; it is also a potent barrier to in vivo administration of other gene delivery systems such as liposomes, murine retrovirus, and various synthetic DNA complexes [87,89].

Furthermore, baculoviruses, similar to other enveloped viruses, are very fragile. The structure of the envelope is essential for the infectivity and transduction capacity of the virus due to the anchored GP64, responsible for the fusion of the viral and cellular membranes [90]. Because of this, the virus is vulnerable to mechanical shear force, resulting in relatively low virus stability. This common problem is also seen in other enveloped viruses, such as retroviruses. Ultracentrifugation is often required for the purification of BV but also leads to significant loss of infectivity, probably due to damage to viral envelopes. The modest thermal stability, coupled with the risk of inactivation by serum complement, could further restrict the in vivo application of baculovirus gene delivery vectors [76,91].

### 7.1. Baculoviral Gene Delivery Vectors

With the discovery of the ability of baculoviruses to transduce mammalian cells, their therapeutic use expanded rapidly. Since then, the viral genome has been modified and manipulated to improve transduction efficiency and simplify its production. Consequently, several vector systems have been developed, including BacMam, Bac-to-Bac, MultiBac, and derivatives of these AcMNPV transfer vectors [92,93,94,95].

For foreign genes to be properly expressed, a viral or mammalian promoter must be chosen and recognized by the transcriptional machinery of the transduced cell. The viral p10 and polyhedrin promoters have been used most frequently to promote transcription in insect cells due to their high expression activity. However, a mammalian promoter can also be used to drive heterogeneous gene expression after viral transduction. These types of systems are usually called BacMam [93].

Some mammalian promoters used in the BacMam system include gene promoters from cytomegalovirus (CMV), simian virus 40 (SV40), chicken beta-actin (CAG), and hepatitis B virus (HBV), among others [96].

Viral and mammalian promoters can be combined with genomic enhancers to promote transgene transcription. Specifically, the insertion of an additional homologous region 1 (*hr1*) into baculoviruses has been used to activate mammalian promoters and results in improved stability and prolonged transgene overexpression [97]. 

The choice of the promoter will also depend on the nature of the gene of interest to be expressed (protein, lncRNA, shRNA, gRNA). Here, we will review the engineering behind the design of baculoviral vectors depending on the nature of the therapeutic gene of interest (GOI) to be used. In addition, we will focus on different strategies for optimizing its use in personalized treatments.

### 7.2. Protein Expression

The most widely used baculovirus-based gene therapy strategy consists of the expression of one or more proteins with a certain biological activity. This biological function can be used as a therapeutic effector against one or more diseases.

Among the many pathologies targeted by gene therapy, cancer is one of the most addressed. Regarding baculovirus-based protein expression against cancer, we can mention some examples; chicken anemia virus apoptin expression against hepatoma [98], herpes simplex virus thymidine kinase expression against glioblastoma [99], expression of an antiangiogenic fusion protein against prostate and ovarian cancer [100], CD40 ligand and IL-15 in bladder tumors [101] and sodium iodide symporter expression in hypopharyngeal carcinoma [102,103]. 

Baculovirus-based protein expression for therapeutic purposes was also implemented for other diseases such as hepatic encephalopathy [104] and cardiovascular diseases [105,106,107,108,109,110,111,112]. Furthermore, this strategy was used also in regenerative medicine and tissue engineering [113,114,115,116,117,118].

### 7.3. shRNA and ncRNA Expression

In addition to the expression of proteins, another therapeutic strategy is the expression of non-coding RNA with the desired biological function.

Transduction of different cell lines with a baculovirus expressing shRNAs (short-hairpin RNAs) knocks down the expression of the target mRNA [119]. Additionally, baculoviruses have been used to mediate RNA interference (RNAi). Recombinant baculoviruses encoding RNAi sequence suppressed target gene expression by 95% in cultured cells and by 82% in vivo in rat brains [87,120]. 

Recently, Gottardo and Pidre et al. developed a recombinant baculovirus targeting the mitochondrial peptide Humanin and its rat homolog Rattin by shRNA expression. Knocking down Humanin expression induced pituitary tumor cells in vitro and in vivo and reduced significatively tumor progression in mice models [121]. The same recBV demonstrated similar effects in ovarian cancer cells [122].

In addition, Chen et al. constructed a hybrid baculoviral system with Sleeping Beauty transposon expressing the long noncoding RNA (LncRNA) PTENP1 for treatment of hepatocellular carcinoma in mice models [123].

In a novel strategy, Li et al. developed a dual baculoviral system in which one baculovirus encodes miR-214 target sequence flanked by loxP sites, and the other virus encodes the Cre recombinase. Co-transduction in adipose-derived stem cells (ASC) led to the generation of a miR-124 sponge. As a result, levels of mi-214 were downregulated and the bone regeneration capacity of ASC cells was improved [124,125]. 

### 7.4. CRISPR/Cas Systems

With the emergence of CRISPR/Cas technologies, extremely useful not only for gene editing but also for epigenetic regulation and RNA targeting, baculovirus-based delivery systems have been upgraded to incorporate it.

Hindriksen et al. developed a Tn7-based recombination system with a transfer vector encoding both sgRNA and sgCas9. The resulting recombinant baculoviruses were able to edit target genes in different mammalian cells [126]. More recently, Aulicino et al. optimized a system based on DNA assembly (MultiMate) to facilitate vector construction and improve BV manufacturing and implemented homology-independent targeted integration (HITI) for precise insertion after spCas9 action [127].

Regarding disease gene therapies, baculovirus-delivered CRISPR/Cas systems were used to treat nerve injuries [128] and to regulate serum cholesterol levels [129] in animal models.

On the other hand, these types of tools have been used in insect systems to efficiently edit leptidopteran genomes [130] as well as to edit baculoviral genomes to improve their bioinsecticide capacity [131].

### 7.5. recAAV Produced in BEVS

AAVs are small non-enveloped icosahedral negative-single-stranded DNA viruses (Parvoviridae). Due to their simplicity, tropism, and the ability to transduce non-dividing cells expressing transgenes long-term, AAV-based viral vectors have been widely used for gene therapy [132,133,134]. Initially, rAAVs were produced by transfecting mammalian cell lines with plasmids carrying the therapeutic sequence and ITRs, plus other helper plasmids with the AAV genes and the necessary ones from other viruses (usually adenovirus genes). This approach did not allow for production in the yield necessary for therapy in humans [135]. Those limitations led to studies that evaluated the rAAV production by complementation systems [136]. This is how BEVS was used for this purpose. First, three rec-baculoviruses were used (rep and cap genes in two independent BVs and the ITR-GOI-ITR construct in the third: the so-called ThreeBac system, Glybera®) [137]. Later, different optimizations were performed including the use of fewer rec-baculoviruses [138,139,140,141]. All these contributions position the baculovirus–insect cell production platform as a suitable alternative for the AAV gene therapy industry.

## 8. Enhancing Baculovirus Transduction Efficiency in Gene Delivery

Several factors contribute to baculovirus transduction efficiency, including cell type, envelope glycoproteins, cell chromatin status, and promoter type, among others. Since we have already discussed strategies to improve viral entry, we will now focus on other aspects related to heterologous construction design for improved and more durable transgene expression.

### 8.1. Promoter Selection

Promoters used in baculovirus gene delivery systems can dictate the efficiency of transduction in gene therapy. The most commonly used viral promoters in insect cell-based expression systems include polyhedrin and p10. If the coding sequence for the heterologous protein is fused to that corresponding to the N-terminal region of gp64, then it can be incorporated into the viral envelope and delivered to target cells. Other viral promoters include p6.9, the viral vp39 promoter, and the immediate early gene (IE1) promoter, which have good levels of expression, particularly at early stages [142,143].

However, if the goal is for a therapeutic gene to be expressed in a mammalian cell, it is necessary to choose a promoter that can be correctly recognized by the cell’s transcriptional machinery. In these cases, the different promoters of the human cytomegalovirus, ubiquitin C, phosphoglycerate kinase, and elongation factor-1 alpha (EF1α) have been incorporated into different baculoviral systems [144]. The efficiency with which the promoter is transcribed will depend to a great extent on the cell type and the physiological state of the cells. Additionally, promoters can be combined with transcriptional enhancers to increase the expression of the transgene [145]. In addition, the expression step of the promoter can also alter gene expression. The numerous combinations of viral and mammalian promoters make the baculoviral gene delivery system an alternative with extremely high versatility and customization capacity [81,96].

### 8.2. Extending Baculovirus GOI Expression In Vivo and In Vitro

As mentioned above, despite their many advantages, recombinant baculoviruses have a relatively short window of transgene expression in vivo of up to 21 days [146]. One reason that could explain this is that baculoviruses activate both the classical pathway as the complement alternative leading to viral inactivation [147]. Several methods have been employed to prevent complement activation and prolong gene expression. Activation of the alternative and classical complement pathway can be prevented by viral envelope surface expression of decay-accelerating factor (DAF), factor H-like protein 1, and C4b-binding protein, among others [148,149]. Kawai et al. concluded that the fusion of the CD46 and CD59 proteins with DAF is capable of protecting viral particles against complement (Figure 2C) [150]. Inhibition of complement activation, through the aforementioned methods, can effectively prolong gene expression in vivo. Although gene expression at short times can be useful for wound repair, other types of therapies such as cancer therapy may require longer gene expression.

The addition of other glycoproteins than GP64 to the baculovirus envelope can extend the window of expression in vivo. Insertion of the vesicular stomatitis virus (VSV) G protein extended gene expression to 178 days in DBA/2J mice and 35 days in BALC/c mice [151,152].

Sung et al. demonstrated that the formation of non-replicating expression episomes, from two different baculoviral vectors (one encoding a recombinase and the other encoding the gene of interest flanked by recombinase-specific recombination sites), leads to an increase in both, as in the duration of the expression of transgenes [153].

Additionally, they have used the same strategy to generate a replicative episome that provides constant gene expression for up to 48 days [154]. Here, the strategy is similar where one vector encodes the Cre recombinase and this cleaves part of the genome of the other vector, Forming an episome that contains the therapeutic gene, the sequence of the origin the sequence of the origin of replication oriP and the coding sequence for the EBV EBNA1 protein capable of recognizing this origin and promoting replication (Figure 2A) [153,154,155].

## 9. Concluding Remarks

Baculoviruses, and especially AcMNPV, have been extensively studied and applied in various biotechnological uses for years. These different efforts in the development of new baculovirus-based technologies have generated different products and proofs of concept in many fields including not only agriculture but also animal and human health [81,96,134].

Genetic engineering of AcMNPV to generate modified virions has been widely used for basic and applied research. These modifications were first based on homologous recombination in insect cell cultures but later, following the generation of AcMNPV bacmids, genomic modification in bacteria was made possible [96]. In addition, novel strategies have been developed for AcMNPV gene modification, including the construction of synthetic genomes [156,157] or gene editing procedures mediated by CRISPR/Cas technologies [127,131].

Due to the versatility of baculoviruses, they can not only be used as biopesticides, but also as vectors for the expression of recombinant proteins and nanostructures such as VLPs, for the generation of protein-display platforms, and as a gene delivery vectors to transduce mammalian cells [134]. The first commercial products reaching the market derived from the use of BEVS for human health were Cervarix® (a human VLP vaccine against HPV; GlaxoSmithKline, 2007); Provenge® (a recombinant protein for prostate-cancer immunotherapy; Dendreon, 2010); Flublok® (a human-subunit vaccine against influenza; Protein Sciences Corporation, 2013); and Glybera® (human gene-therapy product for the treatment of lipoprotein-lipase deficiency based on recAAV produced by BEVS) [134,158]. On the other hand, several products for veterinary use produced in BEVS are already on the market (Table 1). Regarding COVID-19, Novavax Inc., sold today under the names Nuvaxovid and Covovax, was the first BEVS-produced COVID-19 vaccine to gain FDA approval [159].

In recent years, different virus-based products have been approved for use in humans, both for gene therapy and in vaccine formulations [160]. At the same time, basic and applied studies on baculoviruses have exploded, producing more fundamental knowledge and allowing the emergence of better strategies for their modification and application. In consequence, it is very likely that in the coming years the different BV-based technologies could cross the threshold towards clinical trials and begin to be commercialized.

The wide versatility of baculoviruses and their indisputable utility, make them a powerful tool for human and animal health, and the multiple fields of application from the agricultural to the pharmaceutical industry mean that "the magic staff" is no longer just a wordplay, due to the particular shape of the virions, but a proper reference to their enormous technological potential.

## Figures and Tables

**Table 1:** BEVS-based health-related products of interest.

Table	Commercial Name	Manufacturer	Target (Organism/Disease)	Composition	Clinical Stage	Year of Approval */Phase III Trial	Reference
Human vaccines	Cervarix®	GlaxoSmithKline	human papillomaviruses 16 and 18	VLPs of L1 proteins from HPV 16 and 18	Approved	2007 *	[155]
	Flublok® Quadrivalent	Sanofi Pasteur	influenza virus	HA from 4 different strains of influenza A and B	Approved	2016 *	[156]
	Nuvaxoid™/Covovax™	Novavax	SARS-CoV-2	S protein	Approved	2022 *	[157]
	N/A	West China Hospital of Sichuan University	SARS-CoV-2	S RBD (residues 319-545)	Phase III	2021	[158]
	N/A	Sanofi Pasteur/GlaxoSmithKline	SARS-CoV-2	S (pre-fusion state) without its transmembrane domain	Phase III	2021	[159]
	NanoFlu™	Novavax	Influenza virus	HA, NA and M1 proteins	Phase III	2019	[160]
	N/A	Novavax	Influenza viruses and SARS-CoV-2	HA and Spike (recombinant) nanoparticles	Phase I/II	N/A	[161]
Veterinary products	Porcilis Pesti®	MSD Animal Health	Pestivirus C (CSFV) (Classical swine fever)	E2 protein	Approved (withdrawn from UE market)	2009 *	[162]
	Circumvent® PCV-M G2	MSD Animal Health	Porcine circovirus 2, Mycoplasma hyopneumoniae	ORF2, non replicative antigen of Mycoplasma hyopneumoniae	Approved	2013 *	[163]
	Bayovac® CSF-E2	Bayer AG/Pfizer Animal Health	Pestivirus C (CSFV) (Classical swine fever)	E2 protein	Approved	2000 *	[164]
	Ingelvac CircoFLEX®	Boehringer Ingelheim	Porcine circovirus 2	ORF2	Approved	2008 *	[165]
	Circumvent® PCV ^A^	Merck Animal Health	Porcine circovirus 2	ORF2	Approved	2005 *	[166]
	Porcilis PCV® ^A^	MSD Animal Health	Porcine circovirus 2	ORF2	Approved	2005 *	[167]
	INTERDOG™	TORAY Industries, Inc.	Treatment of atopic dermatitis in dogs	Canine gamma interferon	Approved in Japan	2005 *	[168]
	Virbagen® Omega	Virbac	Treatment of infections with feline leukemia virus (FeLV) and/or feline immunodeficiency virus (FIV)	Feline omega interferon	Approved	2001 *	[169]
Gene Therapy	Glybera®	uniQure	Lipoprotein lipase deficiency	Adeno-associated vector with LPL gene	Approved (withdrawn from market in 2017 due to high cost)	2012 *	[170]
Others	Diamyd®	Diamyd	Type I diabetes	GAD65	Phase III	2021	[171]
	Provenge®	Dendreon Pharmaceuticals	Prostate cancer	PAP-GM-CSF fusion protein	Approved	2010 *	[172]

^A^ Same product but licensed under different names depending on geographic region.

## Data Availability

Not applicable.

## Abbreviations

AcMNPV	Autographa californica Multiple Nucleopolyhedrovirus
BEVS	baculovirus-based protein expression system
ODV	occlusion-derived virus
OB	occlusion bodies
BV	budded virus
HA	hemagglutinin
ZIKV	Zika virus
VSV	Vesicular stomatitis virus
VSV-G	Vesicular stomatitis virus glycoprotein
TBV	transmission-blocking vaccines
VLP	virus-like particles
MOI	multiplicity of infection
HIV	Human immunodeficiency virus
SP	signal peptide
TM	transmembrane
CTD	cytoplasmic terminal domain
MHC	major histocompatibility complex
OVA	ovalbumin
PEI	polyethylenimine
PEG	polyethylene glycol
RIPK1	receptor interaction protein kinase 1
AAV	adeno-associated virus
FDA	U.S. Food and Drug Administration
CMV	cytomegalovirus
SV40	simian virus 40
CAG	chicken beta-actin
HBV	Hepatitis B virus
GOI	gene of interest
lncRNA	long non-coding RNA
shRNA	short hairpin RNA
gRNA	guide RNA
ncRNA	Non-coding RNA
RNAi	interference RNA
CRISPR	clustered regularly interspaced short palindromic repeats
EF1α	elongation factor-1 alpha
DAF	decay accelerating factor
EBV	Epstein-Barr virus
